# Proteomics profiling of serum exosomes from azithromycin-sensitive and resistant *Mycoplasma pneumoniae*-infected patients reveals candidate biomarkers for diagnosis

**DOI:** 10.3389/fmed.2025.1677573

**Published:** 2025-12-10

**Authors:** Huifeng Fan, Feng Huang, Chen Chen, Qiaoli Ren, Jing Zhou, Diyuan Yang, Gen Lu

**Affiliations:** 1 Department of Respiration, Guangzhou Women and Children’s Medical Center, Guangzhou Medical University, Guangzhou, Guangdong, China; 2 Department of Respiration, Liuzhou Hospital, Guangzhou Women and Children’s Medical Center, Liuzhou Guangxi, China; 3 Guangzhou Laboratory, The Fifth Affiliated Hospital, Guangzhou Medical University, Guangzhou, Guangdong, China

**Keywords:** *Mycoplasma pneumoniae*, azithromycin resistance, serum exosome, proteomics profiling, diagnosis

## Abstract

**Introduction:**

*Mycoplasma pneumoniae* (*M. pneumoniae*) infections are prevalent among school-age children, and an increasing number of patients are developing resistance to azithromycin (AZM). However, effective biomarkers for diagnosing AZM resistance are currently lacking. This study aimed to identify potential biomarkers for AZM resistance in *M. pneumoniae* infections by analyzing serum exosomes.

**Methods:**

Serum samples were collected from *M. pneumoniae*-infected patients before and after AZM treatment and were categorized into two groups: responders and non-responders. Serum exosomes were isolated and analyzed using nanoparticle tracking analysis (NTA) and proteomics profiling by liquid chromatography-tandem mass spectrometry (LC-MS/MS). Differential protein expression patterns were compared between AZM-sensitive and resistant patients, and potential biomarkers were identified and validated.

**Results:**

Distinct exosomal protein expression patterns were observed between AZM-sensitive and resistant patients. The HIF-1 and IL-17 signaling pathways were found to be associated with AZM resistance. Four proteins (KCTD12, LTF, TF, and MPO) were identified as potential biomarkers for distinguishing responders from non-responders. These biomarkers demonstrated over 80% sensitivity and 73.33% specificity in differentiating between the two groups.

**Conclusion:**

The study successfully identified four potential biomarkers (KCTD12, LTF, TF, and MPO) for AZM resistance in *M. pneumoniae* infections. These biomarkers may serve as useful diagnostic tools in clinical settings, aiding in the identification of patients who may not respond to AZM treatment. Future research should focus on validating these biomarkers in larger cohorts and exploring their potential applications in clinical practice.

## 1 Introduction

*Mycoplasma pneumoniae* (*M. pneumoniae*) is a common cause of community-acquired respiratory tract infections in school-aged children, leading to higher hospitalization rates and a significant burden on families (1, 2). Macrolides, including azithromycin (AZM), are the first-line drugs for patients infected with *M. pneumoniae* due to their low toxicity and the absence of contraindications (3, 4). However, AZM resistance has been observed in some cases, and the number of resistant cases has been increasing worldwide in recent decades (5, 6). A comprehensive understanding of AZM resistance can inform the development of strategies to guide the treatment and diagnosis of *M. pneumoniae*.

The mechanism of azithromycin resistance has been reported to be associated with functional mutation in the 23S rRNA (7, 8), which does not fully explain the principles underlying AZM resistance. The biology governing the response to AZM in *Mycoplasma pneumoniae* remains largely unknown and requires further investigation. The application of multiple omics approaches, such as whole-genome sequencing and proteomic profiling, holds promise for unraveling the complexity of the disease (9–11). With the rapid development of mass spectrometry techniques, protein identification and proteomic profiling have been widely adopted both experimentally and clinically, providing avenues for selecting biomarkers for disease diagnosis, prognosis prediction, and treatment targeting (12–14).

Exosomes are nanoscale vesicles generated through endocytic membrane transport during endosome maturation and secreted into the extracellular environment (15, 16). The abundant cargo proteins, as well as other biological materials in exosomes, manifest diverse functions in different physiological or pathological disease processes (16). Emerging evidence indicates that serum exosomes contain multiple compartments, such as lipid, nucleic acid, and proteins that have been characterized as the favorable biomarkers for disease diagnosis and therapeutics in circulation (17–19). By profiling the proteomics of circulating exosomes from gastrointestinal stromal tumor patients, the biomarkers were successfully identified and exhibited promising efficacy for disease diagnosis, prognosis, and monitoring the tumor response to treatment (20). However, the proteomics profiling of serum exosomes from *Mycoplasma pneumoniae* has not been fully determined. The prognosis prediction and diagnosis function of serum exosomal proteins in *Mycoplasma pneumoniae* for AZM resistance also remains unclear.

## 2 Materials and methods

### 2.1 Study design

In this study, 20 patients with *M. pneumoniae* infection were enrolled between August 2024 and November 2024. The serum samples were collected from the peripheral blood of each patient before and after AZM treatment and subgrouped into responders and non-responders according to the response of respiratory and inflammatory symptoms to the treatment (21). The non-responder was strictly defined as a child who: remained febrile (≥38 °C) for >48 h after the third AZM dose, and showed no improvement in cough, auscultation findings, or chest-X-ray infiltrates by day 3. Conversely, all children who cleared fever, improved symptomatically, and had reduced infiltrates by day 3 were classified as responders. Serum was collected at three strictly defined time-points: baseline (pre-treatment, day 0), and day 3 immediately after the third daily dose (standard 3-day evaluation). For parallel reaction monitoring (PRM) validation experiment, another 23 patients with *M. pneumoniae* infection, consisting of 13 non-responders and 10 responders, were included. To further assess the effectiveness of the candidates, samples from 30 patients were analyzed, consisting of 15 responders and 15 non-responders. All research in this study was conducted in compliance with the ethical guidelines of the Helsinki Declaration and was approved by the Ethics Committee at Guangzhou Women and Children’s Medical Center. The informed consent was signed by all enrolled participants.

### 2.2 Exosome isolation and characterization

First, the collected serum was subjected to centrifugation at 10,000 g for 30 min at 4 °C to remove cell debris. The cell-free serum was collected to isolate exosomes using the Total Exosome Isolation Reagent kit (4478360, Thermo Fisher Scientific). Briefly, serum was added to the Total Exosome Isolation reagent (volume ratio: 5:1). After vortexing, the mixture was incubated for 30 min at 4 °C, followed by centrifugation at 10,000 g for 10 min. Exosomes contained in the pellet were collected and resuspended in 1X PBS by discarding the supernatant. The particle size and concentration of isolated exosomes were measured using nanoparticle tracking analysis (NTA) with ZetaView PMX 110 (Particle Metrix, Meerbusch, Germany) and transmission electron microscopy (TEM). Briefly, the isolated exosome samples were appropriately diluted using 1X PBS buffer to measure the particle size and concentration. NTA measurement was recorded using the ZetaView system. For TEM characterization, exosome-containing pellets were fixed with 2% glutaraldehyde in 0.1 M sodium cacodylate buffer, followed by another fixation using 2% osmium tetroxide. After dehydration, the pellets were embedded in propylene oxide (Electron Microscopy Sciences, Inc.) for the next ultramicrotome section preparation. The particles were examined, and the images were captured by using a digital camera-assembled transmission electron microscopy (Jeol Electric 1400; Jeol Ltd.).

### 2.3 Liquid chromatography-tandem mass spectrometry analysi*s*

The proteomics profiling of serum exosomes was performed by liquid chromatography-tandem mass spectrometry (LC-MS/MS) analysis using a QExactive MS instrument (Thermo Fisher Scientific). Briefly, the peptide library of exosome proteins was prepared using tryptic digestion. Exosome pellets were lysed in 8 M urea/100 mM Tris–HCl pH 8.0, reduced with 10 mM DTT (30 min, 56 °C) and alkylated with 25 mM iodoacetamide (30 min, room temperature, dark). After 4-fold dilution with 50 mM NH₄HCO₃, sequencing-grade trypsin (Promega, 1:50 w/w) was added and digestion proceeded overnight at 37 °C. Peptides were desalted on C18 spin columns (Thermo), dried and re-suspended in 0.1% formic acid for LC-MS/MS. Then, the peptides resuspended in mobile phase A were injected at a flow rate of 300 nL per min, separated by a 132 min gradient of 5–22% acetonitrile (ACN) in mobile phase A, followed by an 18 min gradient of 22–38% ACN in mobile phase A. The general setting for LC-MS/MS: 2.0 kV spray, 275 °C transfer tube; full-MS 350–1,800 *m*/*z*, 70,000 resolution, AGC 5 × 10^4^, 50 ms injection, lock-mass 445.120025; top-20 HCD-MS^2^ (1.4 *m*/*z* isolation, 28% NCE, 17500 resolution, AGC 5 × 10^4^, 50 ms), 25 s dynamic exclusion, unassigned, +1 and ≥+7 charges excluded. Automatic gain control (AGC) was set at 5E4. Fixed first mass was set as 100 *m*/*z*. PRM was used for the verification of candidate biomarkers.

### 2.4 Database mapping

The resulting MS/MS data were processed using MaxQuant search engine (v.1.5.2.8). Tandem mass spectra were searched against human UniProt database concatenated with reverse decoy database to look for human proteins. The mass tolerance for precursor ions was set as 20 ppm in first search and 5 ppm in main search, and the mass tolerance for fragment ions was set as 0.02 Da. Carbamidomethyl on Cys was specified as fixed modification and acetylation modification. Oxidation on Met were specified as variable modifications. FDR was adjusted to <1% and minimum score for modified peptides was set >40.

### 2.5 Identification of differently expressed proteins

Student’s 𝑡-test, and the log2 fold change (log_2_FC) ≥1.5, *p*-value <0.05 were set as the cutoff values for differently expressed proteins (DEPs) searching using the R package DESeq2, and the related volcano plot and heatmap were generated using the R package ggplot. Functions and pathways enrichment for candidate DEPs were analyzed using the online databases the Database for Annotation, Visualization, and Integrated Discovery (DAVID).^1^

### 2.6 Enzyme-linked immunosorbent assay

Human blood samples were collected in tubes containing lithium heparin anticoagulant, kept on ice ≤30 min, then centrifuged (1,800 × g, 15 min, 4 °C) to obtain plasma. Plasma was aliquoted, snap-frozen in liquid N₂ within 1 h of collection, and stored at −80 °C until analysis. Samples were thawed once, vortexed, and spun (10,000 × g, 5 min, 4 °C) to remove cryo-precipitates. The supernatant plasma was collected for enzyme-linked immunosorbent assay (ELISA) analysis. For ELISA analysis, the Human Myeloperoxidase Quantikine ELISA Kit (DMYE00B, RD system), Transferrin Human ELISA Kit (EHTF, Invitrogen), Human BTB/POZ domain containing protein KCTD12 (KCTD12) ELISA Kit (MBS7201044, MyBioSource), and Human LTF/Lactoferrin ELISA Kit (EH309RB, Invitrogen) were performed to detect the serum levels of MPO, TF, KCTD12, and LTF according to the manufacturer’s instructions, respectively.

### 2.7 Statistics analysis

Differences between groups were determined using the two-sided Student’s *t*-test or two-way analysis of variance (ANOVA) test when appropriate. Differences were considered significant when the *p*-value was <0.05. The receiver operating characteristic curve (ROC) was analyzed using the method of Wilson/Brown. Unlike the discovery and PRM phases, fold-change was not used for ELISA validation because the assay’s sole purpose was to establish a diagnostic cutoff and associated sensitivity/specificity; once the optimal threshold is defined, fold-change loses clinical relevance for a binary classifier. All calculations and graphs were generated using GraphPad Prism 8.0.1.3.

## 3 Results

### 3.1 Stratification and clinical characteristics of patients with *Mycoplasma pneumoniae* infection

**Figure 1:**
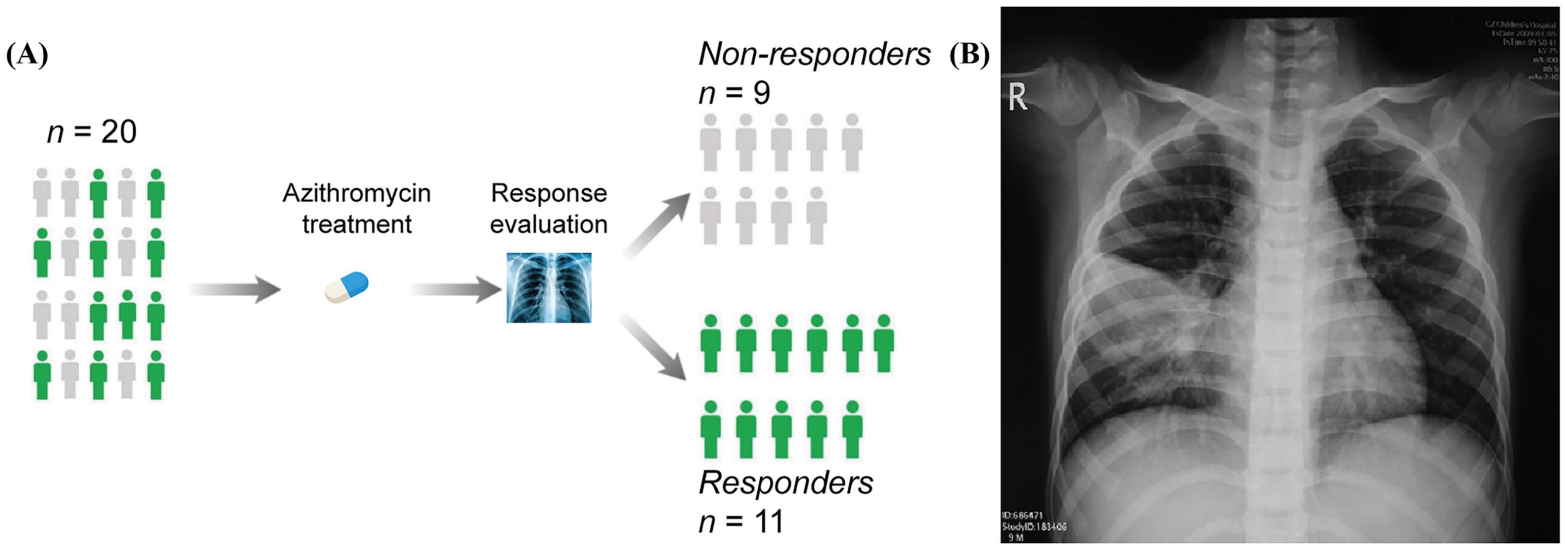
Stratification and clinical characteristics of patients with *Mycoplasma pneumoniae* infection. **(A)** Schematic of treatment strategy and stratification of patients. **(B)** X-ray images show the inflammation foci in the lung and bronchi of responders and non-responders.

Twenty patients with *M. pneumoniae* infection who received a single treatment of AZM were randomly enrolled in this study. The serum samples of the participants were collected before and after AZM treatment for the next exosomes isolation and cargo proteins identification. In line with the previous study (21), responders (R) and non-responders (NRs) are defined by evaluating the response of respiratory and inflammatory symptoms to the treatment, by which 20 patients were divided into 11 responders and 9 non-responders (Figure 1A). The X-ray images showed the shrinkage of inflammation foci in the lungs of the responders, which remains stable in non-responders (Figure 1B). The basal clinical characteristics for the participants are illustrated in Table 1.

**Table 1:** Clinical characteristics of 20 patients with *M. pneumoniae* infection treated with azithromycin.

Characteristic	Responders (*n* = 11)	Non-responders (*n* = 9)	*p*-value
Age	5.78 ± 2.33	6.46 ± 2.66	0.558^a^
Sex			0.658^b^
Male	6 (54.5%)	5 (55.6%)	
Female	5 (45.5%)	4 (44.4%)	
Rale			0.535^b^
Yes	4 (36.4%)	4 (44.4%)	
No	7 (63.6%)	5 (55.6%)	
Fever duration (days)	8.64 ± 1.9	13.67 ± 5.66	0.013^a^
Respiratory rate (bpm)	26.55 ± 6.20	31.33 ± 16.16	0.375^a^
WBC (×10^9^/L)	7.00 ± 2.40	9.756 ± 3.935	0.069^a^
Neutrophils (%)	64.73 ± 11.46	67.00 ± 10.92	0.658^a^
Hs-CRP (mg/L)	31.65 ± 18.08	29.44 ± 14.62	0.771^a^

WBC, white blood cell; HsCRP, high-sensitivity C-reactive protein.

a Student’s *t*-test.

b Chi-squared test.

### 3.2 Proteomics profiling of serum exosomes from patients with *Mycoplasma pneumoniae* infection

**Figure 2:**
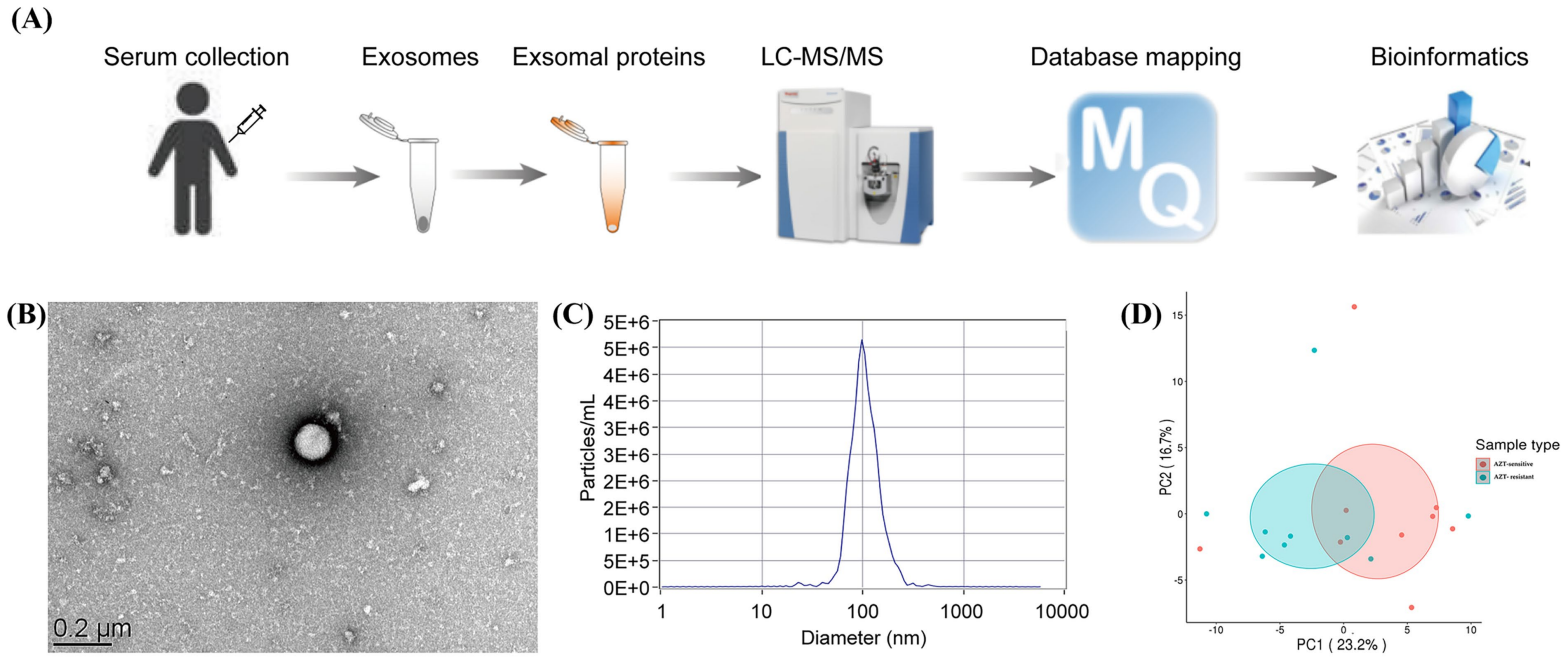
Proteomics profiling of serum exosomes from patients with *Mycoplasma pneumoniae* infection. **(A)** Schematic of proteomics profiling of serum exosomes. **(B)** The morphology of isolated exosomes under an electron microscope. **(C)** The size of isolated exosomes analyzed using nanoparticle tracking analysis (NTA). **(D)** PCA analysis shows the expression pattern of exosomes cargo proteins in between AZM-sensitive and resistant patients, PC1 and PC2 account for 23.2 and 16.7% of the total variance, respectively.

**Figure 3:**
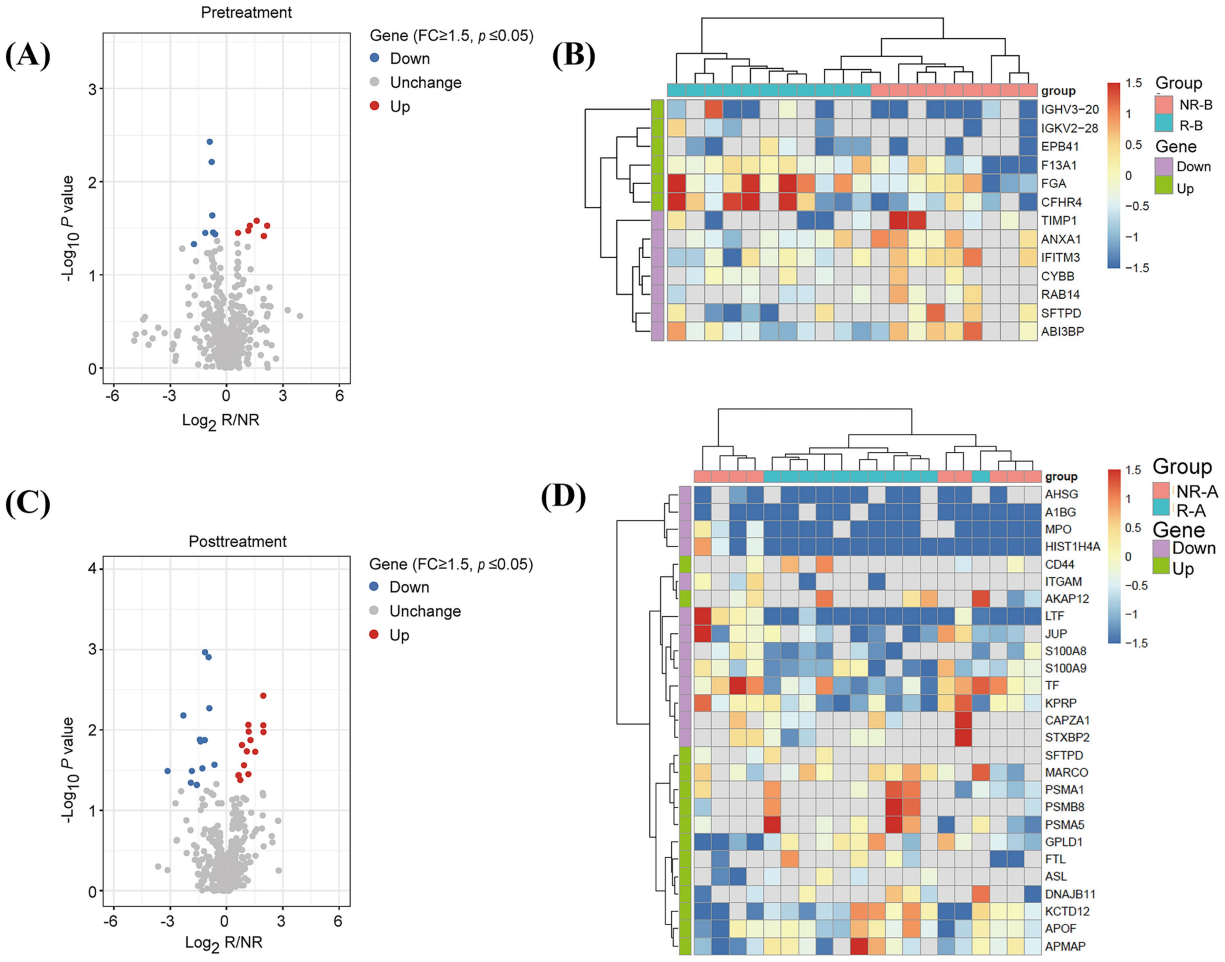
Identification of differentially expressed proteins (DEPs) in exosomes. **(A)** Volcano plot shows the differentially expressed proteins in responders compared to non-responders at the stage of AZM pretreatment. **(B)** Heatmap shows the expressions and clusters of differentially expressed proteins in responders compared to non-responders at the stage of AZM pretreatment. **(C)** Volcano plot shows the differentially expressed proteins in responders compared to non-responders’ posttreatment with AZM. **(D)** Heatmap shows the expressions and clusters of differentially expressed proteins in responders compared to non-responders’ posttreatment with AZM.

To profile the proteomics of serum exosomes from patients, the collected serum samples were subjected to exosome isolation and characterization of the cargo proteins sequentially (Figure 2A and Supplementary Table S1). First, using NTA, it was shown that the concentration of exosome particles was up to 1.2 × 10^10^ particles per one microliter serum sample, and 97.5% of the isolated exosome particles had approximately a diameter of 100 nm (Figures 2B,C), suggesting a reliable resource for the next characterization of the exosome cargo proteins. Next, tandem mass spectrometry (MS/MS) analysis identified a total of 911 proteins, in which 678 proteins are quantifiable in the isolated exosomes from serum samples of 11 responders and 9 non-responders at pretreatment stage. PCA analysis demonstrated a distinguished pattern of protein abundance in exosomes between AZM-sensitive and resistant patients (Figure 2D). Noticeably, 13 DEPs consisting of 6 upregulated and 7 downregulated ones were successfully identified (Table 2 and Figure 3A). Moreover, 27 DEPs, including 13 upregulated and 14 downregulated proteins, were successfully identified in responders compared to non-responders at posttreatment (Table 3 and Figure 3C). Unsupervised hierarchical clustering analysis of DEPs expression showed two distinct gene clusters associated with several cellular transduction signaling (Figures 3B,D).

**Table 2:** Thirteen differentially expressed proteins (DEPs) in exosomes in responders compared to non-responders before azithromycin treatment (R-B/NR-B).

Protein accession	Ratio	Regulated type	*p*-value	Protein name
A0A075B6P5	3.966	Up	0.039	IGKV2-28
A0A0C4DH32	4.469	Up	0.030	IGHV3-20
P00488	1.522	Up	0.036	F13A1
P01033	0.298	Down	0.047	TIMP1
P02671	2.225	Up	0.034	FGA
P04083	0.575	Down	0.006	ANXA1
P04839	0.654	Down	0.037	CYBB
P11171	2.351	Up	0.030	EPB41
P35247	0.455	Down	0.036	SFTPD
P61106	0.588	Down	0.023	RAB14
Q01628	0.535	Down	0.004	IFITM3
Q7Z7G0	0.608	Down	0.035	ABI3BP
Q92496	3.041	Up	0.026	CFHR4

**Table 3:** Twenty-seven differentially expressed proteins (DEPs) in exosomes in responders compared to non-responders after azithromycin treatment (R-A/NR-A).

Protein accession	Ratio	Regulated type	*p*-value	Protein name
P02765	0.382	Down	0.014	AHSG
P02787	0.53	Down	0.005	TF
P02788	0.268	Down	0.045	LTF
P02792	3.912	Up	0.011	FTL
P04217	0.446	Down	0.013	A1BG
P04424	2.269	Up	0.011	ASL
P05109	0.516	Down	0.001	S100A8
P05164	0.113	Down	0.033	MPO
P06702	0.641	Down	0.027	S100A9
P11215	0.278	Down	0.032	ITGAM
P14923	0.334	Down	0.048	JUP
P16070	2.118	Up	0.019	CD44
P25786	2.234	Up	0.036	PSMA1
P28062	3.899	Up	0.004	PSMB8
P28066	3.883	Up	0.009	PSMA5
P35247	1.562	Up	0.037	SFTPD
P52907	0.409	Down	0.030	CAPZA1
P62805	0.203	Down	0.007	HIST1H4A
P80108	1.664	Up	0.042	GPLD1
Q02952	2.419	Up	0.013	AKAP12
Q13790	1.544	Up	0.037	APOF
Q15833	0.372	Down	0.013	STXBP2
Q5T749	0.453	Down	0.001	KPRP
Q96CX2	2.244	Up	0.009	KCTD12
Q9HDC9	1.913	Up	0.028	APMAP
Q9UBS4	2.883	Up	0.019	DNAJB11
Q9UEW3	1.766	Up	0.015	MARCO

### 3.3 Enrichment of the signaling pathways associated with AZM resistance

**Figure 4:**
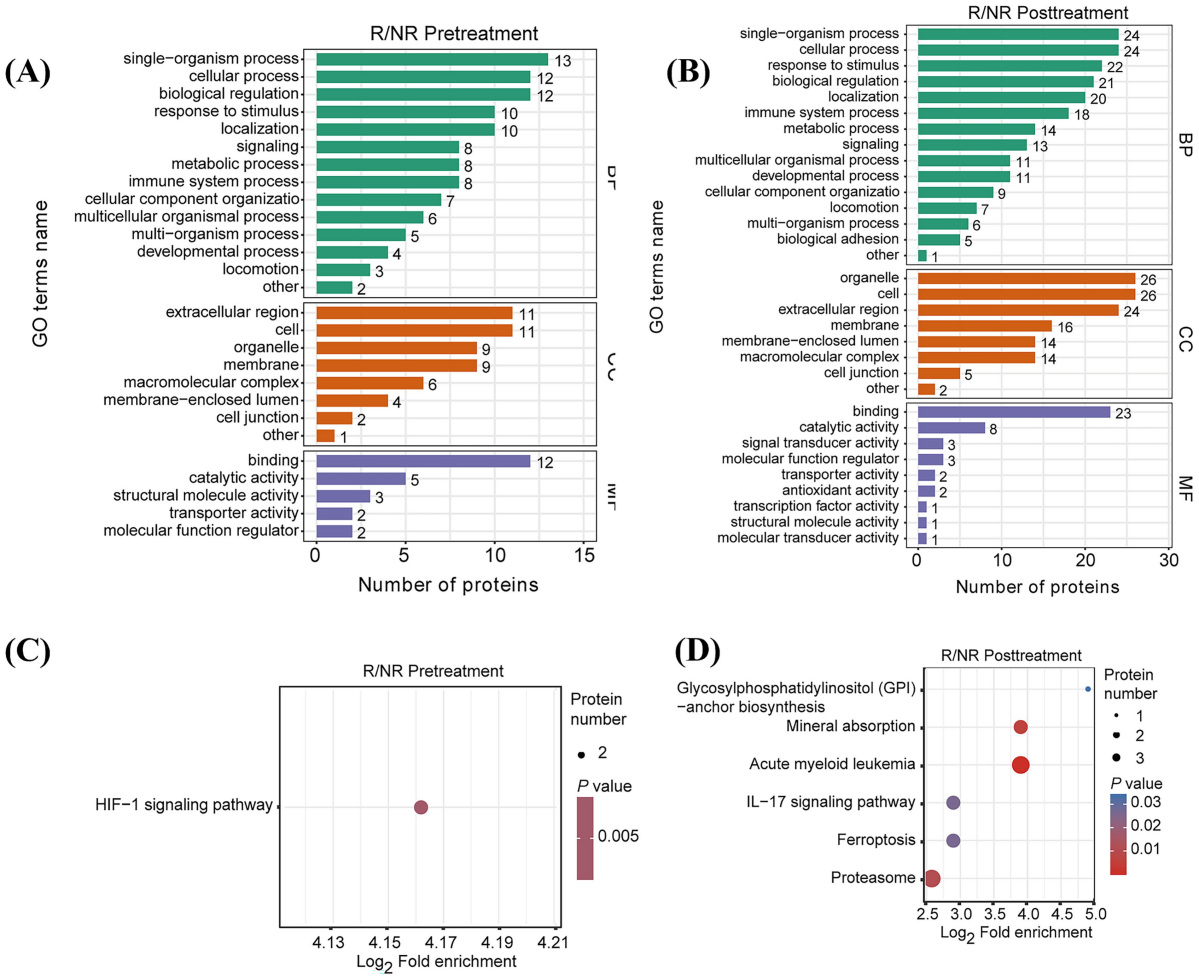
Gene ontology of differentially expressed proteins (DEPs). **(A)** Gene ontology analysis of differentially expressed proteins shows the pathways associated with AZM resistance at the stage of pretreatment. **(B)** Gene ontology analysis of differentially expressed proteins shows the pathways associated with AZM resistance at the stage of posttreatment. **(C)** KEGG analysis of differentially expressed proteins shows the pathways associated with AZM resistance at the stage of pretreatment. **(D)** KEGG analysis of differentially expressed proteins shows the pathways associated with AZM resistance at the stage of posttreatment.

To understand the biological relevance behind AZM resistance, the dysregulated proteins were subjected to gene ontology (GO) and signaling pathway enrichment analysis. GO analysis showed that multiple pathways related to biological process (BP), cellular composition (CC), as well as molecular function (MF) were enriched (Figures 4A,B). Signaling pathway enrichment analysis displayed that the enriched pathways were mainly related to HIF-1 signaling at pretreatment, and IL-17 signaling at the posttreatment stage (Figures 4C,D). Moreover, molecular function-related pathways such as signal transduction, antioxidants and transcription occurred at posttreatment of AZM compared to the pretreatment stage (Figures 4A,B), suggesting a potential association of these pathways with AZM resistance.

### 3.4 Selection of the candidate biomarkers for AZM resistance

To verify the expression of potential biomarker proteins, the top 20 of the dysregulated proteins identified above were selected and qualified in a total of 66 serum exosome samples from 13 responders and 10 non-responders using PRM. Among those, four proteins were successfully quantifiable with at least two unique peptides. Specifically, potassium channel tetramerization domain containing 12 (KCTD12) was significantly upregulated, while transferrin (TF), lactotransferrin (LTF), and myeloperoxidase (MPO) were downregulated at the pretreatment stage. KCTD12 and LTF were significantly upregulated, and TF and MPO were significantly downregulated at the posttreatment stage (Table 4). It was previously reported that the exosome cargo proteins KCTD12, TF, LTF, and MPO acted as the diagnostic biomarkers or therapeutic targets for different types of diseases (20, 22–24), suggesting that these four candidates are detectable in serum and potentially can be applied as non-invasive diagnostic biomarkers.

**Table 4:** Four potential biomarker proteins validated by PRM.

Protein name	R-A/NR-A ratio	R-A/NR-A *p*-value	R-B/NR-B ratio	R-B/NR-B *p*-value
TF	0.56	0.005	0.42	0.002
LTF	1.28	0.025	0.22	0.008
KCTD12	1.44	0.029	2.16	0.006
MPO	0.57	0.022	0.15	0.008

### 3.5 Efficacy evaluation of the candidate biomarkers for AZM resistance

**Figure 5:**
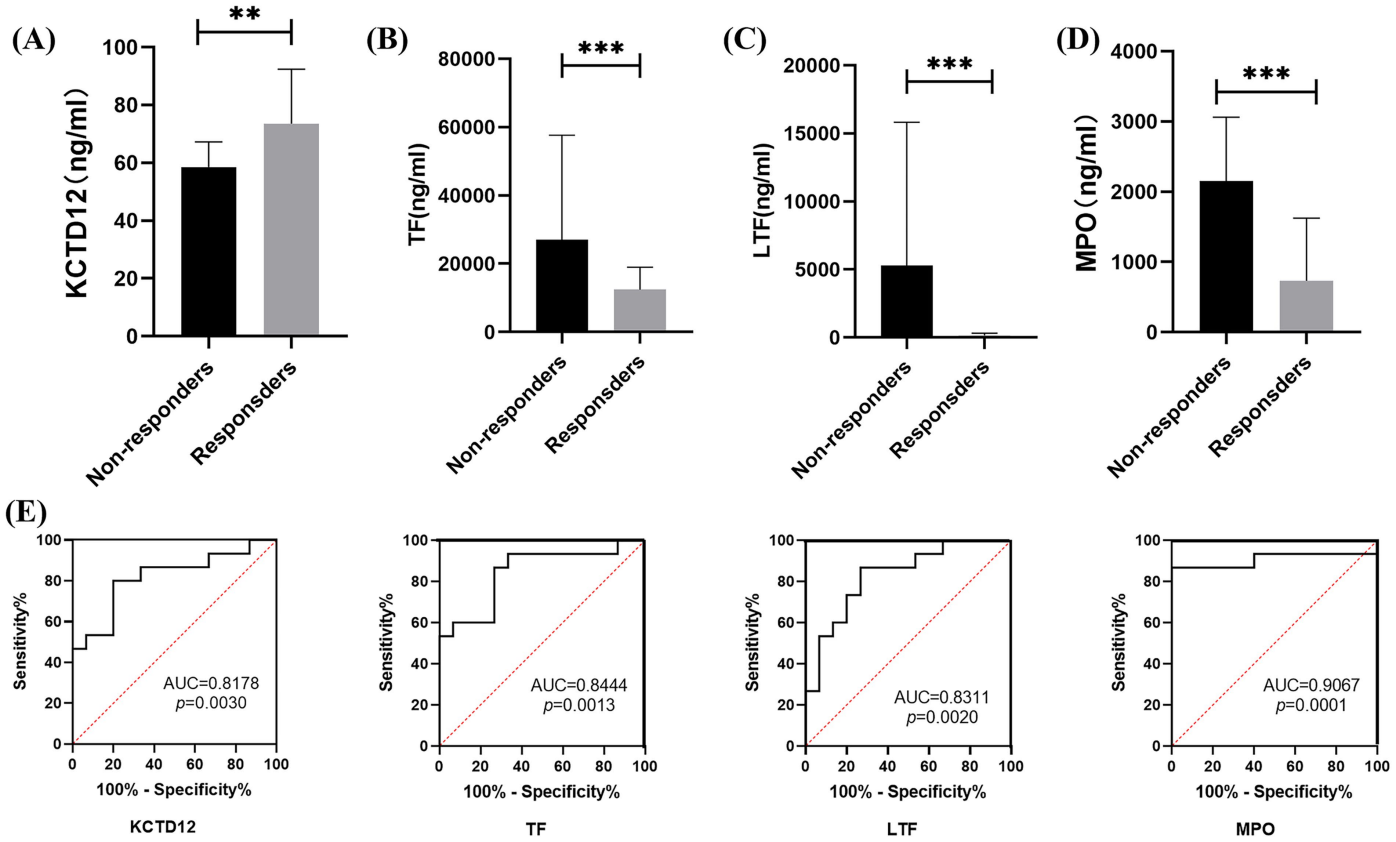
Evaluation of the candidate biomarkers for AZM resistance diagnosis. **(A–D)** The serum levels of KCTD12, TF, LTF, and MPO in responders and non-responders analyzed using ELISA, respectively. Data expressed as mean ± SD, ^*^p<0.05, ^**^p < 0.01, ^***^p < 0.001, and ^****^p < 0.0001. **(E)** The ROC curves for KCTD12, TF, LTF, and MPO in diagnosing AZM resistance.

To evaluate the power of the candidates being utilized to diagnose the AZM resistance, we determined the serum level of KCTD12, TF, LTF, and MPO in 30 patients using ELISA (15 R and 15 NRs), respectively. Before the AZM treatment, the level of KCTD12 was upregulated in the responders compared to the non-responders (Figure 5A), the levels of TF, LTF, and MPO were downregulated in the responders compared to the non-responders (Figures 5B–D). Next, we performed ROC analysis to assess the sensitivity and specificity of these four candidate biomarkers in the prediction of AZM resistance. The cutoff value of KCTD12 was set at 65.9 ng/mL, the sensitivity was 80%, the specificity was 80%, and the area under the curve (AUC) value was 0.8178 to distinguish between the responders and non-responders. The cutoff value of TF was 13,972 ng/mL, the sensitivity and specificity for the prediction of AZM resistance were 86.67 and 73.33%, respectively, with an AUC value of 0.8444. Similarly, the cutoff value of LTF was 222.5 ng/mL, the sensitivity and specificity were 86.67 and 73.33%, with an AUC value of 0.8311, and the sensitivity and specificity of MPO were 86.67 and 93.33%, with an AUC value of 0.9067, and the cutoff value was 906.2 ng/mL (Figure 5E).

## 4 Discussion

*Mycoplasma pneumoniae* (*M. pneumoniae*) is one pathogen of the community-acquired infectious diseases (25). It is largely seen in school-aged young children with the trait of invulnerability to common antibiotic therapy, which is the main reason for morbidity and mortality in young adults (1, 5). AZM therapy shows efficacy in some sensitive patients but fails in the resistant ones (4, 7). Lacking reliable laboratory biomarkers discriminating against the sensitive and resistant patients before treatment makes choosing suitable therapeutic regimen more challenging for pediatricians. In the present study, by profiling the proteomics of serum exosomes, we identified four candidate biomarkers, KCTD12, LTF, TF, and MPO, that effectively distinguish responders from non-responders to AZM treatment with a higher reliability at the pretreatment stage. We stress that KCTD12, TF, LTF, and MPO are host-response proteins; their altered serum-exosomal levels mirror the child’s innate reaction to infection with azithromycin-resistant *M. pneumoniae* and do not reflect or cause the bacterial mutation that directly confers resistance (26, 27). Given the features of friendly-accessible and low invasiveness of collecting serum from peripheral blood, our findings fundamentally provide options to consider for diagnosing AZM resistance before treatment. Moreover, the identification of HIF-1 signaling and IL-17 signaling associated with AZM resistance also sheds light on understanding the molecular mechanisms underlying AZM resistance and developing novel, effective strategies for treating *Mycoplasma pneumoniae*.

Occasionally, *M. pneumoniae* is intrinsically susceptible to macrolide drugs including AZM, which is also the first-line drug for *M. pneumoniae* respiratory tract infections owing to its low toxicity and the absence of contraindication (28). In the present study, we adopted the clinical definition of AZM resistance-persistent fever ≥38 °C and lack of improvement in cough, chest X-ray or CRP within 48–72 h of standard-dose AZM-as the primary criterion (1). However, the occurrence of AZM acquired resistance as well as the difficulty in monitoring the differences in clinical symptoms between the resistant and sensitive cases in a part of patients makes it a significant obstacle for effectively curing *M. pneumoniae* clinically. Therefore, it is necessary to open new avenues to search for novel biomarkers with the ability to effectively recognize the resistant and sensitive cases at the pretreatment stage, resulting in a relatively precise medication for *M. pneumoniae*. Proteomics profiling has been recently established as a popular avenue to screen biomarkers and therapeutic targets in several diseases (29, 30). Exosomes containing an abundance of cargo proteins exhibit great potential in disease diagnosis and therapy (15, 31, 32). In our current study, we focused on serum exosomes to profile the proteomics that exhibit a distinct protein expression pattern in between the responders and non-responders of AZM treatment, suggesting the complexity of the mechanisms behind the AZM resistance.

In light of the complexity, we sought to search for several biomarkers to predict the response of patients to AZM ahead of treatment by leveraging the proteomics data. It showed that the level of KCTD12 was upregulated, the levels of TF, LTF, and MPO were downregulated in the responders compared to the non-responders, suggesting a potential activity being the diagnostic biomarkers. KCTD12 is deregulated in a variety of different cancer types and associated with cancer malignancy, tumorigenicity, and stemness (33–35). It is also identified as an exosome cargo protein in gastrointestinal stromal tumor (20), suggesting the possibility of being diagnostic biomarkers. In the present study, being the diagnostic biomarker, KCTD12 provides a high sensitivity and specificity for predicting the AZM resistance in patients with *M. pneumoniae* infection. MPO is a lysosomal protein most abundantly expressed in neutrophils and produces hypohalous acids to act their antimicrobial activity (36). It was first identified in the present study as an exosome cargo protein and upregulated in patients with AZM resistance. TF is a blood-plasma glycoprotein that plays a central role in iron metabolism and is responsible for ferric-ion delivery (37, 38). It is a serum exosome-associated protein applicable for diagnosis and treatment in several diseases (39, 40). Moreover, LTF is an iron-binding multifunctional protein with antimicrobial properties by sequestering free iron (41, 42). Collectively, it suggested that the dysregulation of LTF and TF in *M. pneumoniae* infection could not only be the diagnostic biomarkers for AZM resistance, but also highlighted the association of iron metabolism and AZM resistance.

## 5 Conclusion

In conclusion, our study first profiled the proteomics of serum exosomes in AZM-resistant and AZM-sensitive patients with *M. pneumoniae* infection. We found that HIF-1 and IL-17 signaling pathways were associated with AZM resistance. Four serum exosomal proteins, KCTD12, LTF, TF, and MPO, were identified as potential diagnostic biomarkers capable of effectively distinguishing responders from non-responders to AZM treatment, highlighting their promise as non-invasive biomarkers for clinical diagnosis.

## Data Availability

The original contributions presented in the study are publicly available. This data can be found here: accession number IPX0014288000, https://www.iprox.cn/page/ProjectFileList.html?projectId=IPX0014288000.

## Footnotes

^1^ https://david.ncifcrf.gov/
